# Micropattern platform promotes extracellular matrix remodeling by human PSC‐derived cardiac fibroblasts and enhances contractility of co‐cultured cardiomyocytes

**DOI:** 10.14814/phy2.15045

**Published:** 2021-10-07

**Authors:** B.N. Napiwocki, A. Stempien, D. Lang, R.A. Kruepke, G. Kim, J. Zhang, L.L. Eckhardt, A.V. Glukhov, T.J. Kamp, W.C. Crone

**Affiliations:** ^1^ Department of Biomedical Engineering University of Wisconsin‐Madison Madison Wisconsin USA; ^2^ Wisconsin Institute for Discovery University of Wisconsin‐Madison Madison Wisconsin USA; ^3^ Department of Medicine Division of Cardiovascular Medicine University of Wisconsin‐Madison Madison Wisconsin USA; ^4^ Engineering Mechanics Program University of Wisconsin‐Madison Madison Wisconsin USA; ^5^ Department of Cell and Regenerative Biology University of Wisconsin‐Madison Madison Wisconsin USA; ^6^ Department of Engineering Physics University of Wisconsin‐Madison Madison Wisconsin USA

**Keywords:** cardiac fibroblast, cardiomyocyte, extracellular matrix, human pluripotent stem cell, micropattern

## Abstract

In native heart tissue, cardiac fibroblasts provide the structural framework of extracellular matrix (ECM) while also influencing the electrical and mechanical properties of cardiomyocytes. Recent advances in the field of stem cell differentiation have led to the availability of human pluripotent stem cell‐derived cardiac fibroblasts (iPSC‐CFs) in addition to cardiomyocytes (iPSC‐CMs). Here we use a novel 2D in vitro micropatterned platform that provides control over ECM geometry and substrate stiffness. When cultured alone on soft micropatterned substrates, iPSC‐CFs are confined to the micropatterned features and remodel the ECM into anisotropic fibers. Similar remodeling and ECM production occurs when cultured with iPSC‐CMs in a co‐culture model. In addition to modifications in the ECM, our results show that iPSC‐CFs influence iPSC‐CM function with accelerated Ca^2+^ transient rise‐up time and greater contractile strains in the co‐culture conditions compared to when iPSC‐CMs are cultured alone. These combined observations highlight the important role cardiac fibroblasts play in vivo and the need for co‐culture models like the one presented here to provide more representative in vitro cardiac constructs.

## Graphical abstract

For the first time, human PSC‐derived cardiomyocytes and cardiac fibroblasts are co‐cultured on a micropatterned platform which results in enhanced contractility and accelerated Ca^2+^ transient rise‐up time of the cardiomyocytes. The micropattern platform controls the alignment of cardiomyocytes as well as the migration and extracellular matrix remodeling by cardiac fibroblasts. Biomimetic platforms with multiple cell sources will enhance understanding of cardiovascular physiology and allow for more relevant in vitro cardiac constructs.

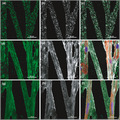

## 1 INTRODUCTION

Cardiovascular disease remains the number one cause of death in the world, despite remarkable advancements in medical, surgical, and device therapies. Development of improved cardiovascular therapeutic strategies will benefit from more representative model systems to test small molecules or biologics. Murine models have provided valuable insights, but it is well known that cardiovascular pathophysiology and drug response in humans can be markedly different from mouse (Kaese & Verheule, 2012; Wehrens et al., 2000). This, combined with high costs and ethical concerns involving the use of large animals, has pushed the field to develop more accurate in vitro models that better recapitulate human cardiovascular pathophysiology and pharmacology.

With the advent of cardiomyocytes derived from human pluripotent stem cells (iPSC‐CMs) we now have a supply of human cardiac cells that can be used in in vitro models (Zhang et al., 2009). The conventional approach for two‐dimensional (2D) culture of iPSC‐CMs is to culture them in monolayers on glass or plastic substrates (Bizy et al., 2013; Lee et al., 2012); however, this method fails to reproduce the highly organized structure typical of cardiovascular tissue. To more accurately mimic the aligned, architectural cardiac phenotype observed in vivo, we previously engineered a 2D in vitro substrate platform that includes physiological substrate stiffness with a 15° chevron micropatterned ECM (Napiwocki et al., 2020). This controlled microenvironment produces aligned, electrically coupled iPSC‐CMs that exhibit anisotropic electrical impulse propagation within a platform suitable for microscopy techniques, allowing for structural, electrical, and mechanical analyses.

After recapitulating cardiac anisotropy in a reproducible manner, we aimed to further enhance this platform with the addition of cardiac fibroblasts to capture some of the diverse, heterocellular composition of native myocardium (Baudino et al., 2006). Cardiac fibroblasts are known to modulate cardiomyocyte mechanical and electrical function in both normal and diseased hearts through the release of paracrine factors, direct cell–cell interactions and cell interactions with the ECM (Liau et al., 2017; Souders et al., 2009). This bidirectional cross talk is especially apparent during cardiac remodeling in which fibrosis can impair cardiac conduction generating a vulnerable substrate for arrhythmias in various forms of heart disease (Pellman et al., 2016; Porter & Turner, 2009). Thus, heterocellular in vitro models that can control myocyte–fibroblast interactions while maintaining the spatial arrangement of the cells will provide a better understanding of myocyte–fibroblast communication and serve as useful tools for high‐throughput drug screening and therapeutic validation. This is particularly true as cardiac fibroblasts are also targets for commonly used cardiovascular drugs including angiotensin receptor blockers/angiotensin converting enzyme inhibitors and β‐adrenergic receptor blockers (Crabos et al., 1994; Meszaros et al., 2000).

In this study, we provide a 2D micropatterned platform for the co‐culture of iPSC‐CMs with iPSC‐CFs. Unlike previous 2D co‐culture models, the platform system presented here better controls myocyte‐fibroblast interactions while also producing an aligned cardiac construct. In addition, due to the recent establishment of iPSC‐CF differentiation protocols (Zhang et al., 2019), our platform represents the first micropatterned co‐culture platform to utilize iPSC‐CMs and iPSC‐CFs. iPSC‐CFs cultured on micropatterned lanes and the 15° chevron pattern remodel the underlying ECM and produce fibers of collagen and fibronectin parallel to the feature direction. When co‐cultured together on the 15° chevron pattern, iPSC‐CMs maintain their alignment and ECM is produced by iPSC‐CFs. Fluorescent optical mapping analysis of electrical activity demonstrates that anisotropic impulse propagation is preserved in the co‐culture conditions as seen previously within cultures containing only iPSC‐CMs (CM Only cultures) (Napiwocki et al., 2020). While conduction velocities remain similar for CM Only and co‐culture conditions, the calcium release time is significantly shorter in the co‐culture conditions and more comparable to in vivo measures. Lastly, we demonstrate the utility of the platform in providing mechanical output data which shows higher contractile strains in the co‐culture models compared to when iPSC‐CMs are cultured without iPSC‐CFs. These results highlight the influence non‐myocyte cell types have on myocyte behavior and why their inclusion is important in order to ensure more accurate in vitro cardiac models.

## 2 METHODS

### 2.1 iPSC cardiomyocyte differentiation

Using a modified version of the small molecule Wnt signaling method, the GiWi protocol (Lian et al., 2013), a human‐induced pluripotent stem cell (iPSC) line iPS‐DF19‐9‐11T (male) (WiCell) was differentiated into cardiomyocytes. A schematic depicts the experimental timeline in Figure 4a. Briefly, iPSCs were seeded on Day 5 in a six‐well plate containing 83 μg/well Matrigel (Corning, qualified by WiCell) at a density of two million cells/well in mTeSR1 medium (STEMCELL Technologies) supplemented with 10 μM ROCK inhibitor (Y‐27632) (Tocris)and cultured with mTeSR medium (STEMCELL Technologies). The iPSCs were fed with mTeSR1 medium every day until 100% confluency was achieved, typically 5 days after the initial seed. On Day 0, the medium was changed to R/B‐ medium: RPMI 1640 medium (Thermo Fisher) and B‐27 Minus Insulin (Thermo Fisher). On Day 0, the R/B‐ was supplemented with 12 µM of the GSK inhibitor CHIR99021 (Biogems) and 1 µg/mL Insulin (Sigma). On Day 1, the medium was exchanged for fresh R/B‐ medium. On Day 3, the medium was changed to R/B‐ medium containing 7.5 µM of the Wnt inhibitor IWP2 (Biogems). On Day 5, the medium was aspirated and cells were fed with fresh R/B‐ medium. On Day 7, and every 2 days thereafter, the cells were fed with RPMI 1640 medium (Thermo Fisher) supplemented with B‐27 Serum‐Free (Thermo Fisher). Beating cells were typically observed between Day 9 and 12 of differentiation. iPSC‐CMs were frozen down in fetal bovine serum (FBS, Invitrogen) containing 10% DMSO (Sigma) and stored in liquid nitrogen on Day 15.

### 2.2 iPSC cardiac fibroblast differentiation

Human cardiac fibroblasts were differentiated from a human‐induced pluripotent stem cell (iPSC) line iPS‐DF19‐9‐11T (male) (WiCell) as described previously (Zhang et al., 2019). Briefly, iPSCs were dissociated with 1 ml/well Versene solution (Invitrogen) at 37℃ for 5 min, and seeded on Matrigel (GFR, BD Biosciences) ‐coated six‐well plates at the density of 2 × 10^6^ cells/well in mTeSR1 medium supplemented with 10 μM ROCK inhibitor (Y‐27632) (Tocris). Cells were cultured for 5 days in mTeSR1 medium with medium change daily until 100% confluence was reached and differentiation was started (day 0). At Day 0, the medium was changed to 2.5 ml RPMI+B27 without insulin and supplemented with 12 µM CHIR99021 (Tocris) and cells were treated in this medium for 24 h (day 1). At Day 1 medium was changed to 2.5 ml RPMI+B27 without insulin (Invitrogen) and cells were cultured in this medium for another day (day 2). At Day 2, the medium was changed to 2.5 ml of the defined fibroblast culture medium (CFBM) (Zhang et al., 2019) supplemented with 75 ng/ml bFGF (WiCell). Cells were fed every other day with CFBM supplemented with 75 ng/ml bFGF and cultured until day 20 for flow cytometry analysis and subculture of cardiac fibroblasts.

### 2.3 PDMS substrate preparation

Polydimethylsiloxane (PDMS) substrates with tunable mechanical properties were created by blending two types of commercially available PDMS types, Sylgard 184 elastomer and Sylgard 527 gel (Dow Corning). Briefly, Sylgard 184 was made by mixing 10 parts base to 1 part curing agent by weight. Sylgard 527 was prepared by mixing equal weights of part A and part B. After both types of Sylgard 184 and 527 were prepared individually, they were then combined in different mass ratios and mixed for 5 min with a glass stir rod, as previously reported (Napiwocki et al., 2020; Palchesko et al., 2012). For this study, PDMS substrates with a Young's modulus of 10 kPa were utilized, which corresponds to 1:50 ratio of Sylgard 184:527. After the PDMS blends were mixed together, they were then poured into a 100‐mm diameter Petri dish and cured overnight at 60℃. To overcome challenges associated with handling low‐modulus PDMS substrates, the PDMS was cured on top of a foundation layer of Sylgard 184 with a final thickness of 2 mm. Once cured, the PDMS substrates were removed from the Petri dish and cut into 1 cm squares with a razor blade and UV sterilized prior to the transfer of ECM.

### 2.4 ECM transfer to patterned and monolayer substrates

The 15° chevron pattern was made as previously described (Napiwocki et al., 2020). Briefly, PDMS (Sylgard 184) was poured on top of a patterned silicon wafer (FlowJEM) and cured at 60℃ overnight to produce a reverse replica of the silicon wafer. Once removed from the silicon wafer, the PDMS was cut into individual stamps which were coated with ECM proteins at 37℃ overnight (83 μg/ml Matrigel, 8 μg/cm^2^ Collagen I (Corning), 5 μg/cm^2^ Fibronectin (Sigma)).

Microcontact printing on soft PDMS substrates was achieved utilizing a sacrificial polyvinyl alcohol (PVA) film method (Jiwlawat et al., 2019; Notbohm et al., 2019; Yu et al., 2012). Briefly, 0.5 g of PVA (Sigma Aldrich) was dissolved in 10 ml of deionized water and air dried overnight in a 100‐mm diameter Petri dish. After the water evaporated, the remaining PVA film was removed from the Petri dish and cut into rectangles slightly larger than the PDMS stamps. The ECM‐coated PDMS stamps were then placed on top of the rectangular PVA films. To help facilitate the transfer of ECM proteins from the PDMS stamp to the sacrificial PVA film, a glass slide and 50 g weight were added on top of the PDMS stamp to apply an even pressure distribution. After 1 h, the PVA film was removed from the PDMS stamp and then brought into conformal contact with the soft PDMS substrate. After 30 min, the substrate was washed with PBS to dissolve the sacrificial PVA film, leaving behind the patterned proteins which were then seeded with cells.

To coat the monolayer substrates with ECM for control samples, a 1‐cm diameter PVC tube was placed on top of the soft PDMS substrates and 83 μg/ml Matrigel was added inside the PVC tube.

### 2.5 iPSC‐CM thawing, purifying, and seeding onto soft PDMS substrates

Figure 4a is a schematic of the experimental timeline used in this study. Cryopreserved Day 15 iPSC‐CMs were thawed onto Matrigel coated 6 well plates at a density of 2.5 million cells/well and fed with EB20 medium: DMEM/F12 (Invitrogen), 20% FBS (Invitrogen), NEAA (Invitrogen), GlutaMax (Lifetech), and 2‐Mercaptoethanol (Sigma). To purify iPSC‐CMs the cells were fed with lactate medium for 10 days (Tohyama et al., 2013) from Day 17–27. The lactate medium consisted of RPMI‐glucose (Life Technologies), B27‐complete (Life Technologies), and 5 mM lactate (Sigma). After 10 days of purification the medium was changed to EB2 medium: DMEM/F12 (Invitrogen), 2% FBS (Invitrogen), NEAA (Invitrogen), GlutaMax (Lifetech), and 2‐mercaptoethanol (Sigma). On Day 29, the EB2 medium was aspirated and exchanged for fresh EB2 medium. CM purities of greater than 95% after lactate purification were confirmed via flow cytometry (Napiwocki et al., 2020). On Day 30, purified iPSC‐CMs were dissociated and singularized with TyrpLE 10× (Lifetech) and seeded onto both patterned and monolayer substrates using EB20 medium, referred to as Day 0 for the experimental time point. The patterned substrates were seeded at a density of 2668 CMs/mm^2^ (95,000 CMs/35.605 mm^2^). This density ensured complete pattern coverage by the cells, while low enough to allow for CM hypertrophy. To control cell attachment, a 1‐cm PVC tube was placed on top of the PDMS substrates and the cell suspension was added inside the tube. The PVC tube was removed the following day for the CM Only and CF Day 0 conditions and removed on Day 5 for the CF Day 4 condition after iPSC‐CFs had been added. Cells preferentially attached to the patterned ECM and do not readily attach to the 10 kPa PDMS substrate. After seeding, the medium was changed to EB2 medium and exchanged every 2 days during the length of culture.

### 2.6 iPSC‐CF seeding and DiI labeling

iPSC‐CFs were maintained in FibroGro plus 2% FBS medium (Millipore) on tissue culture plastic. iPSC‐CFs were then treated with TrypLE Express (Lifetech) for 3 min and seeded onto both Matrigel‐coated PDMS and patterned PDMS at a density of 280 iPSC‐CFs/mm^2^ using EB20 medium. Once attached to PDMS substrates, the iPSC‐CFs were maintained in EB2 medium. In DiI labeling experiments, iPSC‐CFs were treated with 5 μM DiI (Invitrogen) for 30 min prior to being seeded on the PDMS substrates.

### 2.7 Decellularization of PDMS substrates

To more clearly visualize the ECM, PDMS substrates were decellularized as reported previously (Xing et al., 2014). Briefly, samples were washed with PBS prior to the addition of the first decellularization solution (1 M NaCl, 10 mM Tris, and 5 mM EDTA; Sigma) and then placed on an orbital shaker for 1 h at room temperature. The samples were again washed with PBS and placed in a second decellularization solution (0.5% SDS, 10 mM Tris, and 25 mM EDTA; Sigma) and shaken for 10 min at room temperature on an orbital shaker. After a PBS wash, the sample was rinsed in DMEM medium with 20% FBS for 48 h at room temperature and rinsed again with PBS prior to fixing and staining.

### 2.8 Immunofluorescence and image analysis

Samples for each condition were washed once with PBS and fixed in 4% paraformaldehyde (PFA) (Electron Microscopy Sciences) for 15 min at room temperature and then washed with PBS. To permeabilize the cell membrane, the cells were treated with 0.1% Triton (Sigma) for 10 min at room temperature. The cells were then washed with PBS and treated for 30–60 min with a blocking solution: PBS, 2% FBS, 0.1% Triton, 11.2 mg/ml glycine (Sigma), and 50 mg/mL BSA (Sigma). After decellularization, blocking was performed with 5% nonfat dry milk (Bio‐Rad) in 0.2% Triton X‐100 solution and incubated for 1.5 h at room temperature on a rotator followed by two washes with PBS (Zhang et al., 2019). Primary antibodies were diluted in blocking solution and left on the sample overnight at 4℃. The following day the samples were washed with PBS and then the secondary antibodies were applied in blocking solution for 1 h. After application of the secondary antibodies, the samples were washed with PBS and then phalloidin conjugated to tetramethylrhodamine B isothiocyanate (TRITC) (Sigma) was applied at a 50 mg/ml concentration to label actin filaments and DAPI was applied at a 1:1000 dilution to label nuclei. Samples were washed with PBS and transferred to coverslips, where they were mounted using ProLong Gold Antifade (Life Technologies). Primary antibodies used include alpha‐actinin (1:250 dilution, Abcam), N‐cadherin (1:250 dilution, BD Biosciences), Clone TE‐7 (1:100 dilution, Millipore), collagen 1a (1:250 dilution, Santa Cruz), and fibronectin (1:250, Santa Cruz). Secondary antibodies include Alexa Fluor (H+L) 488 goat anti‐mouse, Alexa Fluor 647 (H+L) goat anti‐mouse, Alexa Fluor 488 (IgG1) goat anti‐mouse, and Alexa Fluor 647 (IgG2b) goat anti‐mouse (Invitrogen). A Nikon A1RSi Confocal Microscope with an attached Photometrics CoolSNAP HQ2 camera was used to image the samples.

After image acquisition of fixed samples, the scanning gradient Fourier transform (SGFT) open source MATLAB software was used to provide unbiased quantification of both ECM organization and cytoskeletal alignment (Salick et al., 2020). Because the resolution of the image impacts the quality of the results, only high resolution images captured with a 60× or 100× oil‐immersion lens were used for analysis. The following parameters were used as SGFT inputs on images with a resolution of 4096 × 4096 pixels: 0.21 µm/pix for 60x images and 0.03 µm/pix for 100× images, a pattern size of 2 and a scan resolution of 16.

### 2.9 CaT optical mapping

A high spatial (60 μm/pixel) and temporal (1000 fps) resolution CMOS camera ULTIMA‐L system (SciMedium) was used for fluorescent optical mapping of Ca^2+^ transients (CaT) of the pattern cell preparations stained with Ca^2+^‐sensitive probe (Rhod‐2 AM: Ex/Em: 550/582 nm). After incubation of Rhod‐2 AM for 15 min, spontaneous rates were captured at 37℃ for each condition and ranged from 14 to 41 bpm with no significant difference among groups. After spontaneous rates were captured, all further data collection was then conducted under 1 Hz pacing at 37℃. Fluorescent signals were collected and filtered by a 590 ± 15 nm bandpass filter. A custom‐developed algorithm implemented in MATLAB was then used to analyze the acquired fluorescent signals produced by the samples. CaT duration at 80% relaxation (CaTD) was calculated. Ca^2+^ activation maps were constructed from the activation time sequence, which was determined using the dCa/d*t*_max_ definition. The reconstructed activation map was used to analyze conduction velocity.

### 2.10 Contractility and strain analysis

To assess mechanical function of the patterned constructs, bright field videos of cells spontaneously contracting were acquired at various time points during culture using Nikon‐D elements software and a Nikon DS‐Qi1Mc camera with a frame rate of 18.9 fps (Supplemental Video 6). All samples were maintained at 37℃, 5% CO_2_ and 20% O_2_ using a Tokai Hit environmental chamber. The mechanics analysis was done using an open source software with additional modifications, as described previously (Napiwocki et al., 2020). The open source digital image correlation software, Fast Iterative Digital Image Correlation (FIDIC) (Bar‐Kochba et al., 2015), was used to calculate displacements, *U_x_* and *U_y_*. The FIDIC input parameters were a target subset size of 32 pixels and subset spacing of 8 pixels. The FIDIC outputs were then used to calculate a full field in plane normal and shear strains, as well as principal strains, ε*_1_* and ε*_2_*, for each time point throughout the contraction event (Supplemental Video 7) (Notbohm et al., 2019). The field of view during relaxation was used as the reference frame. A binary mask was then used to eliminate data from areas not occupied by cells and then contractile strain analysis was performed. The second principal strain (i.e., contractile strain) values were averaged for each frame of the video and the maximum of this averaged value over the course of the contraction event (i.e., maximum contractile strain) was used as a measure for comparison between sample conditions.

### 2.11 Statistical analysis

Sample sizes and *p* values are reported in each figure legend and statistical analyses were performed using JMP statistical software (SAS Institute Inc). In the figures, unless otherwise noted, graphs show mean ± standard deviation of the mean. When experiments involved only a single pair of conditions, statistical differences between the two sets of data were analyzed with a two‐tailed, unpaired Student's *t*‐test assuming unequal variances. For data sets containing more than two samples, one‐way ANOVA with a post hoc Tukey's tests were used to determine adjusted *p* values.

## 3 RESULTS

### 3.1 iPSC‐CF ECM remodeling

A well‐known function of CFs is ECM deposition and remodeling which contributes to the highly organized architecture of cardiac muscle. To determine if this function is recapitulated in vitro, we cultured iPSC‐CFs on 10 kPa PDMS substrates micropatterned with lanes of Matrigel. Matrigel is a heterogeneous mixture of ECM proteins including, but not limited to, collagen, fibronectin, and laminin. After 2, 5, and 8 days, the cultures were decellularized in order to more clearly visualize the underlying ECM, and a custom open source MATLAB SGFT software (Salick et al., 2020) was used to quantify the orientation and percent alignment of ECM proteins. In the absence of cells, collagen and fibronectin in Matrigel self‐segregate into local, globular regions when coated on the 10 kPa PDMS surface while laminin is relatively uniformly distributed (Figure S1).

**FIGURE 1:**
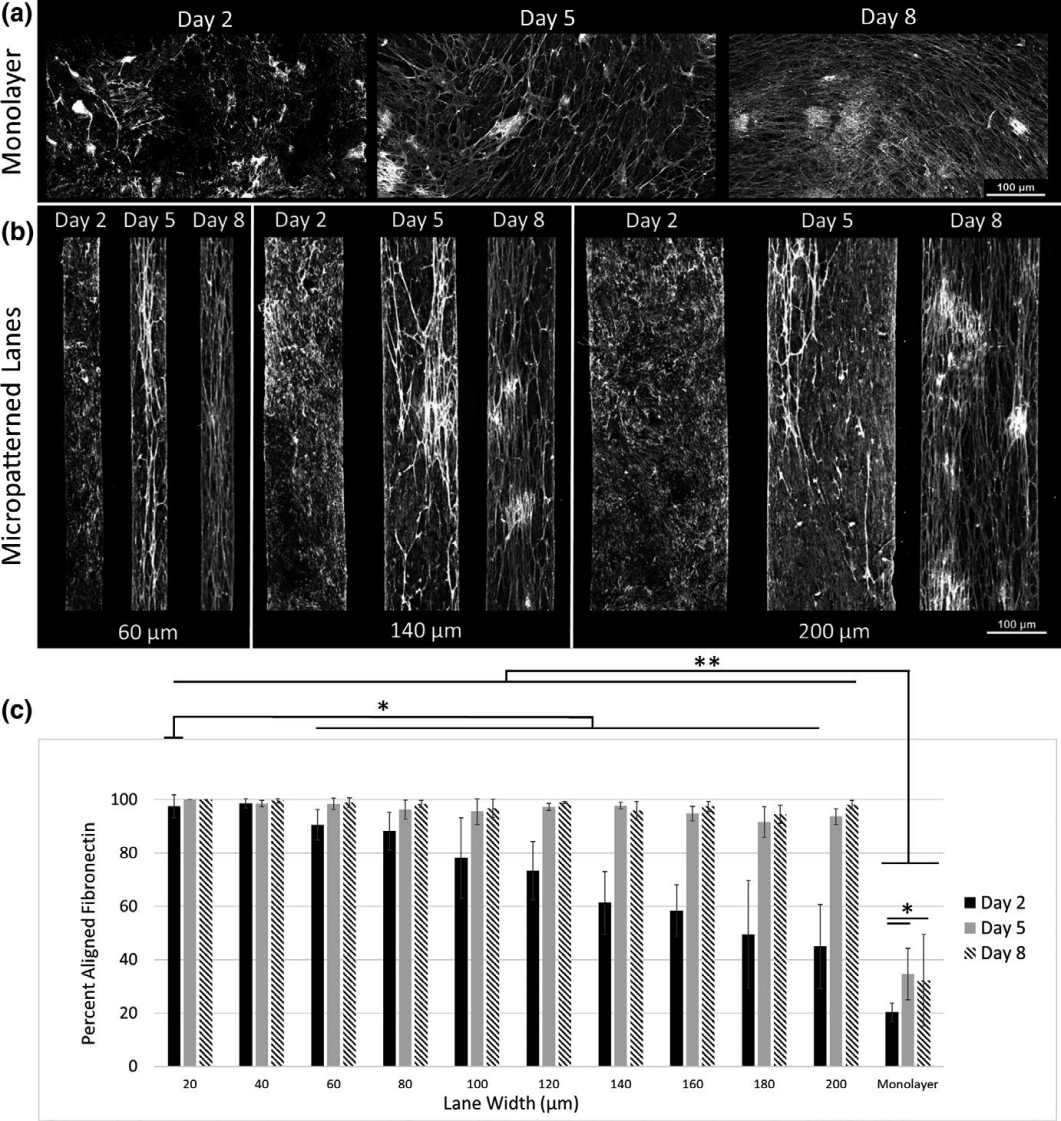
Decellularized fibronectin from (a) monolayers and (b) micropatterned lanes of varying widths after 2, 5, and 8 days of culture with iPSC‐CFs. (c) Quantification of the amount of fibronectin fibers aligned within ±10% of the superior angle using SGFT software. White = Fibronectin. Scale bar = 100 µm. *p < 0.05, **p < 0.001, two‐way ANOVA with post hoc Tukey tests. *n* = 8 (ROIs within a sample) for each day and lane width tested

In the unpatterned, monolayer condition the iPSC‐CFs were able to remodel the globular collagen and fibronectin into fibers after 2 days of culture (Figure 1a). With increased culture time the fibers extended in length and more area of the monolayer control was occupied by ECM fibers. While the fibers were aligned locally there was no global organization of the fibers in the monolayer condition. The amount of global alignment determined by SGFT had a modest improvement from 20% to 32% when comparing decellularized cultures from Day 2 and Day 8, respectively (*p* < 0.05) (Figure 1c). In contrast, the iPSC‐CFs cultured on micropatterned lanes produced highly aligned ECM at all time points relative to the monolayer unpatterned condition. Furthermore, after 2 days of iPSC‐CF culture on micropatterned lanes ECM alignment was inversely correlated with lane width, namely smaller lane widths (those closer to 20 µm) promoted higher alignment than larger lane widths (those closer to 200 µm) (Figure 1b). With extended culture from 2 to 8 days, all lane widths had greater than 95% fiber alignment and were significantly more aligned than the Day 8 monolayer condition (Figure 1c; *p *< 0.001).

**FIGURE 2:**
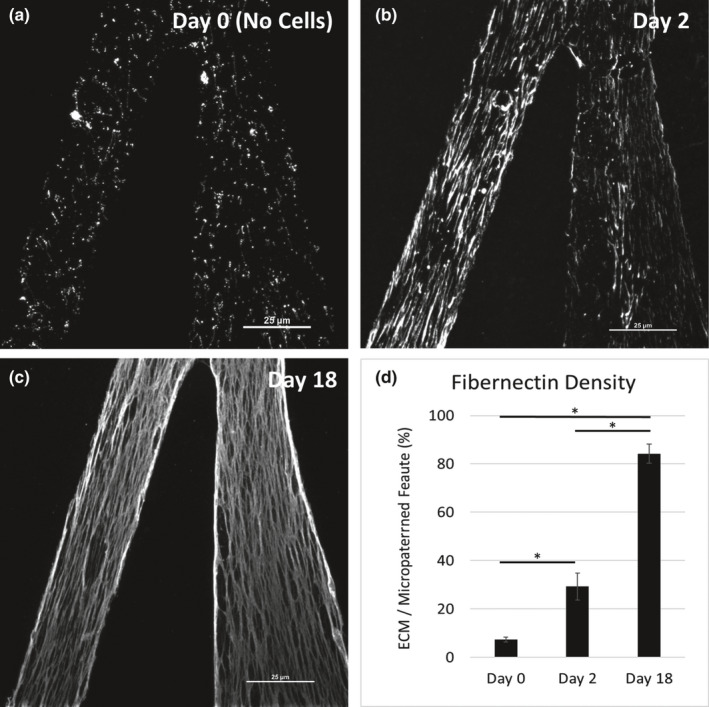
The 10 kPa PDMS substrates patterned with Matrigel using the 15° chevron pattern and stained for fibronectin (white). (a) Day 0 control sample with no iPSC‐CFs attached. Decellularized scaffolds after iPSC‐CFs were cultured for (b) 2 days and (c) 18 days. Scale bars = 25 µm. (d) Quantification of the amount of fibronectin present over the available micropattern area. *p < 0.001, one‐way ANOVA with post hoc Tukey tests. *n* = 6 (ROIs within a sample) for all conditions

The ECM in native heart tissue is anisotropically organized. To recapitulate this in vitro we investigated how ECM is remodeled over time when iPSC‐CFs adhere to and migrate on the 15° chevron pattern composed of 30 µm lanes and bridges (Napiwocki et al., 2020). Like the monolayer Matrigel‐coated condition with no cells seeded (Figure S1), collagen and fibronectin appear globular when Matrigel is applied to the 15° chevron pattern (Figure 2a; Figure S2). When iPSC‐CFs were cultured on the 15° chevron pattern, ECM fibers were produced parallel to the direction of the patterned features (Figure S2). To visualize the remodeled ECM we again decellularized the 15° chevron pattern using the technique described above. After 2 days of culture the iPSC‐CFs had started to remodel the ECM into fibronectin fibers (Figure 2b). Although initially the fibronectin fibers were short and did not persist the length of lanes, within 18 days the fiber bundles were continuous throughout the length of the patterned features and the area density of fibronectin increased greater than 10‐fold, from 7 ± 1% to 29 ± 5% and 84 ± 4% for Days 0, 2, and 18, respectively (Figure 2b–d, p < 0.001). Collagen and laminin were also remodeled after 18 days of culture with iPSC‐CFs (Figure S3). In order to determine if the ECM proteins were remodeled or newly secreted, constructs were patterned with either collagen or fibronectin alone (Figure S4). After 18 days of culture with iPSC‐CFs the constructs were decellularized and stained for collagen and fibronectin. As seen in Supplemental Figure S4, both fibrous collagen and fibronectin were present indicating the iPSC‐CFs synthesized new ECM proteins and remodeled the existing underlying ECM protein.

### 3.2 Micropattern‐guided iPSC‐CF migration

**FIGURE 3:**
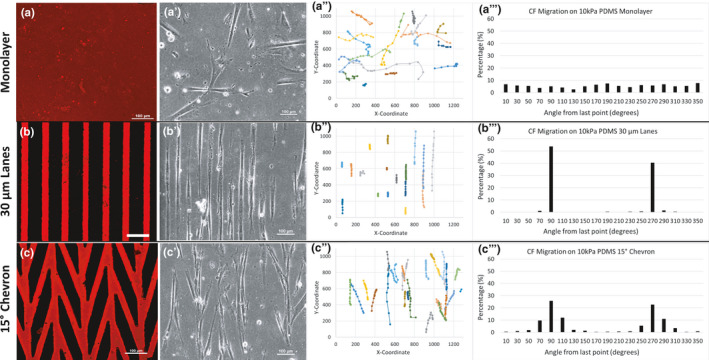
iPSC‐CFs attached to 10 kPa PDMS substrates for 1 day prior to 13 h time‐lapse imaging. Available ECM for cell migration on the 10 kPa PDMS (a) Monolayer, (b) 30 µm lanes and (c) 15° chevron pattern (Laminin = red). (a’–c’) Representative bright field images, (a’’–c’’) migration patterns of individual iPSC‐CFs and (a’’’–c’’’) angle measurements for each of the three conditions tested: monolayer, 30 µm Lanes and 15° chevron pattern. Scale bars = 100 µm for a–c. Units are in µm for a’’–c’’. 90° indicates an upward movement and 270° a downward movement from the last measurement for a’’’–c’’’. Migration patterns of 30–50 iPSC‐CFs were tracked over multiple samples for each condition

The formation of extended networks of ECM fibrils during time in culture suggested that the iPSC‐CFs exhibit migratory behavior in addition to ECM remodeling. We hypothesized that the boundaries of the micropattern lanes influence the migration direction of iPSC‐CFs and thus their ability to remodel ECM locally and globally. We followed the movements of individual iPSC‐CFs on Matrigel‐coated 10 kPa PDMS substrates to characterize the role of cell migration in ECM remodeling using time‐lapse imaging and CellTracker (Piccinini et al., 2016) (Figure 3a–c). When presented with a monolayer of Matrigel on a 10 kPa PDMS substrate, iPSC‐CFs show no orientation preference and migrate in all directions equally (Supplemental Video 1; Figure 3a–a’’’). In contrast, when iPSC‐CFs were cultured on 30 µm micropatterned lanes their migration was limited primarily to movements in the lengthwise direction of the micropatterned lanes denoted by 90 and 270° from the angle of the last measurement (Supplemental Video 2; Figure 3b–b’’’). Due to the close spacing of the micropatterned lanes, iPSC‐CFs were also observed to bridge laterally between adjacent lanes, although this was less common than the migration along the lane length. Next, iPSC‐CFs were presented with a 15° chevron pattern. Similar to the isolated 30 µm lanes, iPSC‐CFs attached only on the micropatterned areas and migrated in the direction of the micropatterned lanes and bridges with no migration observed in areas void of ECM (Supplemental Video 3; Figure 3C–C’’’). There was no statistical difference when comparing the speed of iPSC‐CFs on the monolayer (0.33 ± 0.24 µm/min), 30 µm lane (0.28 ± 0.19 µm/min), and 15° chevron pattern (0.31 ± 0.13 µm/min) substrates. These results demonstrate the iPSC‐CF migration direction can be controlled by micropatterning‐specific geometries on soft 10 kPa PDMS substrates and suggest that the migration of iPSC‐CF determines the orientation of the remodeled ECM fibrils.

### 3.3 Pattern CM‐CF co‐culture

After defining the behavior of iPSC‐CFs and iPSC‐CMs (Napiwocki et al., 2020) when cultured alone on the 15° chevron pattern, we next sought to develop a co‐culture model. Multiple ratios of CM:CF were used including 100:3, 100:6, 100:10, 100:15, and 100:25 in order to determine which ratio best augmented the pattern. We define augmentation as the remodeling of the 15° chevron pattern in a way that preserves cellular attachment and anisotropic alignment while also allowing for growth of cardiac cell types beyond the micropatterned features. The sparsely seeded iPSC‐CFs in the 100:3 ratio resulted in low augmented co‐cultures with similar pattern fidelity as the CM Only condition (100:0) after 18 days in culture (Figure S5). Conversely, iPSC‐CFs in the 100:25 ratio quickly overgrew the pattern and merged adjacent lanes resulting in high augmented patterns, but ultimately lead to pattern deterioration and detachment after 18 days in culture (Figure S5). A middle CM:CF ratio of 100:10 resulted in high‐pattern augmentation while also maintaining iPSC‐CM and iPSC‐CF cellular attachment (Figure S5). This CM:CF ratio of 100:10 is used in the co‐culture studies below and matches ratios used in 3D collagen gel compaction studies (Zhao et al., 2019) as well as the density of iPSC‐CFs used in the migration and ECM remodeling studies described above.

**FIGURE 4:**
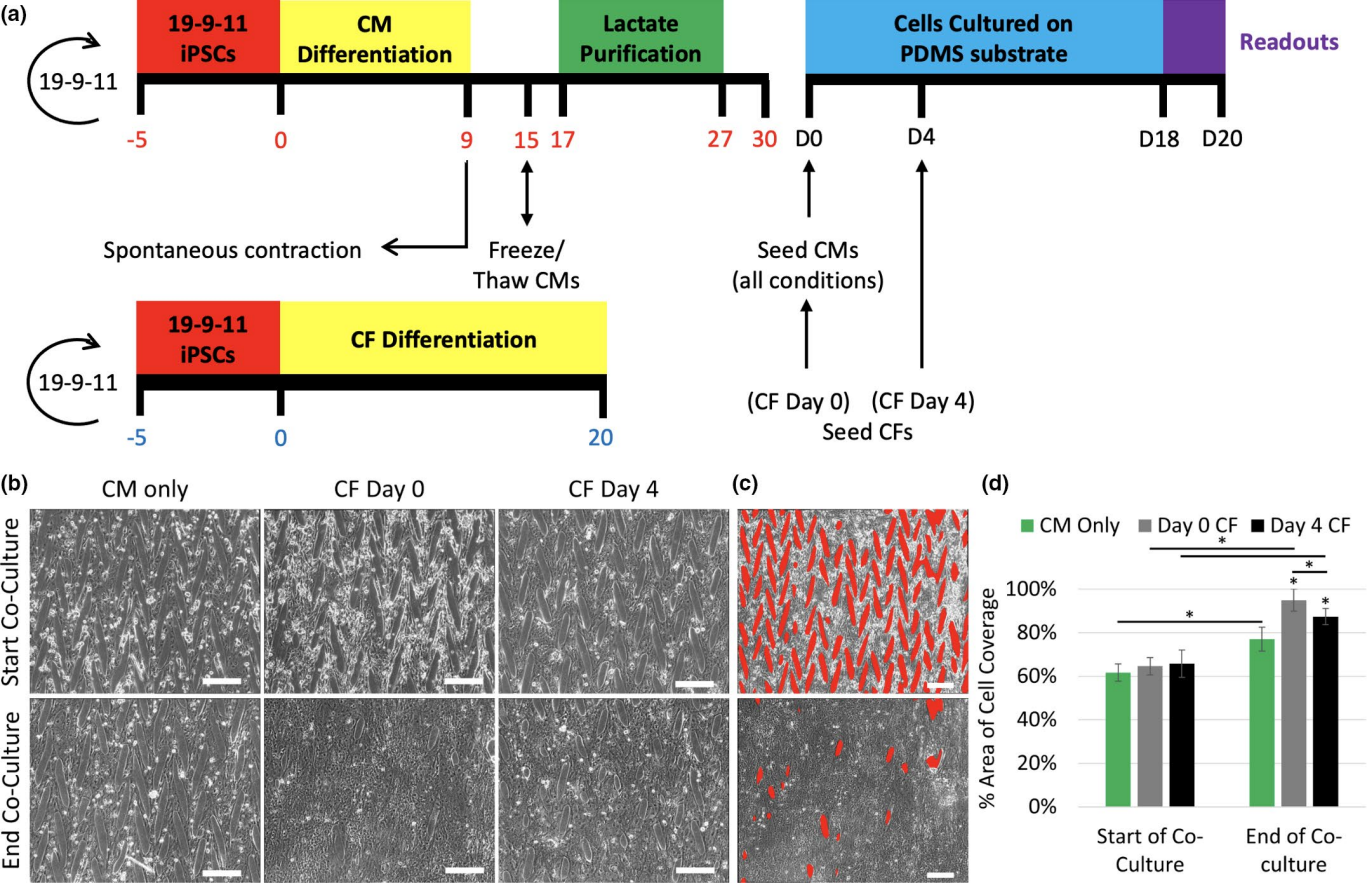
(a) Schematic of the experimental timeline and iPSC differentiation protocols. Red numbers denote days after initiation of iPSC‐CM differentiation, blue numbers are days after initiation of iPSC‐CF differentiation and black numbers represent experimental time points. Co‐culture conditions consist of iPSC‐CF addition on either Day 0 (CF Day 0) or Day 4 (CF Day 4) and are co‐cultured with iPSC‐CMs until Day 18. (b) Bright field images of patterned iPSC‐CMs at the start and end of co‐culture. Bright field locations are the same for the start and end of co‐culture. (c) ROIs of areas not occupied by cells highlighted in red for the start and end of co‐culture. (d) Quantification of area of cell coverage for the start and end of co‐culture. Scale bars = 250 µm. *p < 0.001, two‐way ANOVA with post hoc Tukey tests. *n* = 9 for CM Only, *n* = 11 for Day 0 CF, and *n* = 11 for Day 4 CF (2–3 images per sample, 4 independent experiments)

To identify the optimal time to introduce iPSC‐CFs in co‐culture, iPSC‐CFs were either (1) combined with iPSC‐CMs on Day 0, or (2) on Day 4 after CMs had already been patterned in comparison to control iPSC‐CM Only cultures (Figure 4a). In the Day 0 CF condition, the iPSC‐CMs and iPSC‐CFs initially confined themselves to the micropatterned lanes after 1 day of co‐culture (Figure 4b); however, with extended time in culture the cells migrated and merged adjacent lanes resulting in highly augmented patterns, with little of the underlying 15° chevron pattern still visible. In contrast, the CM Only condition displayed little merging of adjacent lanes during the 18 days of culture. Like the CM Only and Day 0 CF conditions, cells in Day 4 CF were also constrained to the micropatterned lanes after 1 day of co‐culture. The Day 4 CF condition showed some pattern augmentation with the formation of merged lanes but also had areas where the 15° chevron pattern remained visible after 18 days of co‐culture (Figure 4b). Quantification of cell area coverage at the start and end of co‐culture confirmed bright field observations (Figure 4c,d). Namely, the CM Only group showed the smallest pattern augmentation after 18 days with 15.35% more of the pattern occupied by cells (*p *< 0.001), followed by 21.69 (*p *< 0.001) and 30.36% (*p *< 0.001) for the Day 4 CF and Day 0 CF conditions, respectively.

### 3.4 iPSC‐CF attachment, migration, and ECM production in pattern co‐culture

**FIGURE 5:**
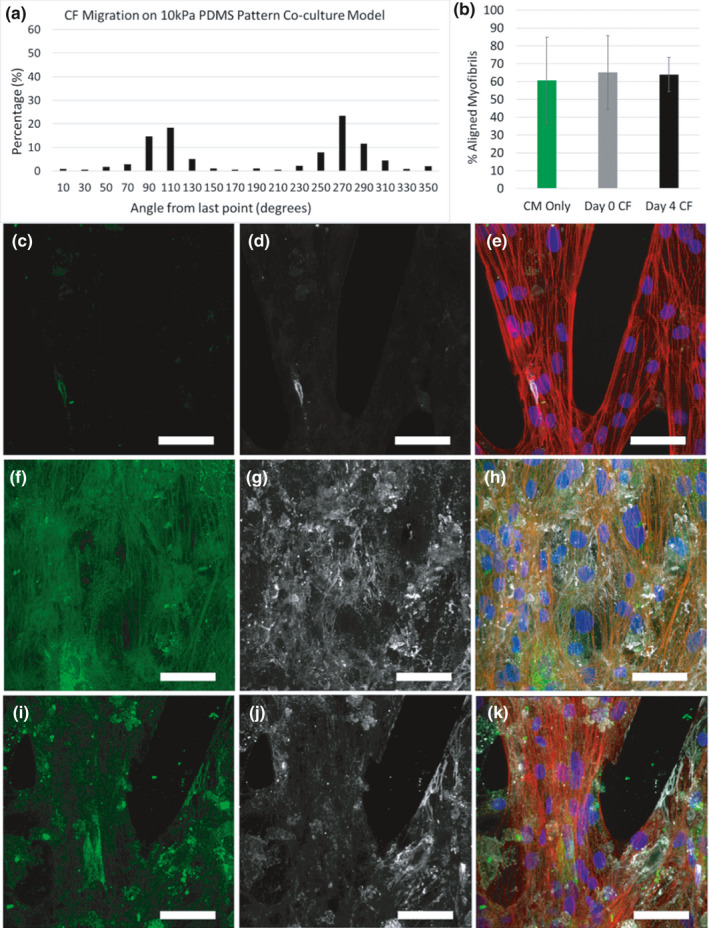
(a) Migration patterns of individual iPSC‐CFs when co‐cultured with iPSC‐CMs on the 15° chevron pattern. 90° indicates an upward movement and 270° a downward movement from the last measurement. Migration patterns of 33 iPSC‐CFs were tracked over two independent experiments. (b) Quantification of the percent of myofibrils aligned within 10° of the superior angle for the three conditions tested determined by SGFT. *n* = 4 (2 ROIs within a sample, 2 independent samples). Collagen and fibronectin expression after 18 days of culture for the CM only (c–e), Day 0 CF (f–h) and Day 4 CF conditions (i–k). Collagen = green, Fibronectin = white, Actin = red, DAPI = blue. Scale bars = 50 µm

To visualize attachment and integration, iPSC‐CFs were stained with DiI prior to co‐culture with iPSC‐CMs. After 1 day of co‐culture, the same number of iPSC‐CFs were observed in the Day 0 CF and Day 4 CF conditions (*p* = 0.155, Figure S6). In order to determine the migration pattern of the iPSC‐CFs in the co‐culture conditions, samples were tracked for 13 h using time‐lapse imaging. Similar to when seeded alone on the 15° chevron pattern (Figure 3c–3c’’), iPSC‐CFs in both co‐culture conditions were influenced by the micropatterned features and migrated along the edges of lanes (Supplemental Video 4; Figure 5a), although iPSC‐CFs migrating on top of the iPSC‐CMs would be more difficult to observe. DiI‐labeled iPSC‐CFs in the middle of merged regions of the patterns were less motile (Supplemental Video 5). To determine how many fibroblasts had integrated and remained at the end of co‐culture, samples were fixed at Day 18 and fibroblasts were identified with the TE‐7 antibody (Kaaya et al., 1995). As expected, the CM Only group had no iPSC‐CFs present (Figure S7a–a’). After 18 days of culture iPSC‐CFs had integrated and augmented both the Day 0 CF and Day 4 CF conditions (Figure S7). As observed during live time‐lapse imaging, Day 18 IHC showed iPSC‐CFs on top of and alongside iPSC‐CMs both in the Day 0 and Day 4 CF co‐culture conditions (Figure S8).

With pattern augmentation occurring in the co‐culture conditions, we next assessed if myofibril alignment was maintained in the co‐culture conditions compared to CM Only in which no augmentation occurred. While myofibril alignment was expected in areas of high‐pattern fidelity, we also found that myofibrils were aligned in areas of the co‐culture conditions that were visibly merged and augmented (Figure S9). For the Day 0 CF and Day 4 CF conditions, the amount of aligned myofibrils was 65.16 ± 20.61% and 63.95 ± 9.48%, respectively, which is in close agreement with 60.63 ± 24.25% reported for the CM Only condition (Figure 5b).

Next, we looked at the ECM present after 18 days of culture. As expected, the CM Only condition displayed little collagen and fibronectin, while both the Day 0 CF and Day 4 CF conditions showed an abundance of these ECM proteins (Figure 5c–k). Decellularization of these tissues after 18 days of culture also confirmed a large amount of ECM remodeling and deposition in the co‐culture conditions compared to the CM Only condition which showed little remodeling and no discernable deposition of ECM (Figure S10). These results highlight the ability of the underlying ECM pattern to influence myofibril alignment in unison with further ECM remodeling and deposition within a iPSC‐CF co‐culture.

### 3.5 CaT properties of pattern co‐culture

**FIGURE 6:**
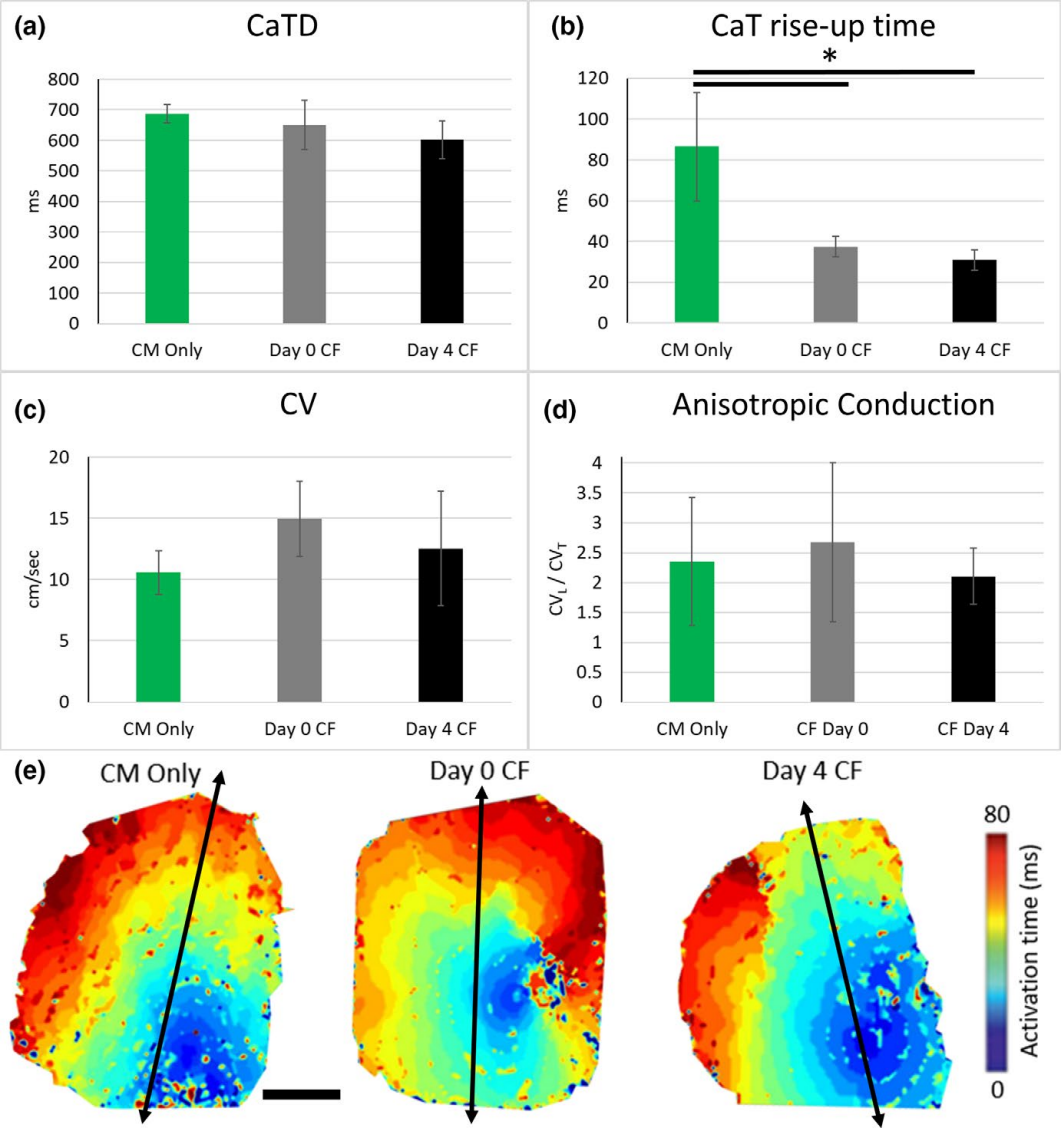
Optical mapping results for (a) CaT duration (CaTD), (b) CaT rise‐up time, (c) conduction velocity (CV) and (d–e) anisotropic conduction velocity for the different conditions tested. Black arrows in optical mapping images represent the major axis of the 15° chevron pattern, scale bar = 2.5 mm. *p < 0.001, one‐way ANOVA with post hoc Tukey tests. *n* = 7 for all conditions (2–3 samples per experiment, three independent experiments)

To determine if iPSC‐CFs co‐cultured with iPSC‐CMs have an impact on iPSC‐CM function in the micropatterned platform, we first assessed intracellular CaT using optical mapping. There was no significant difference in CaT duration (CaTD) between the three conditions tested (CM Only 687 ± 29 ms, Day 0 CF 651 ± 80 ms, Day 4 CF 601 ± 62 ms; Figure 6a). Co‐culture of iPSC‐CMs with iPSC‐CFs accelerated the CaT rise‐up times (37, 30, and 86 ms for the Day 0 CF, Day 4 CF, and CM Only conditions, respectively [*p *< 0.001; Figure 6b]). There was no significant difference in conduction velocity for CM Only (10.5 ± 1.7 cm/s) compared to the Day 0 CF (14.9 ± 3 cm/s) and Day 4 CF (12.5 ± 4.6 cm/s) (Figure 6c). Lastly, although the iPSC‐CFs had augmented the pattern in the co‐culture conditions, the anisotropic conduction velocity observed in CM Only was recapitulated in both patterned co‐culture conditions (Figure 6d,e). The micropatterned platform produced a global conduction velocity orientation maintained throughout the mapped area (Figure 6e).

### 3.6 Contractile strain in pattern co‐culture

**FIGURE 7:**
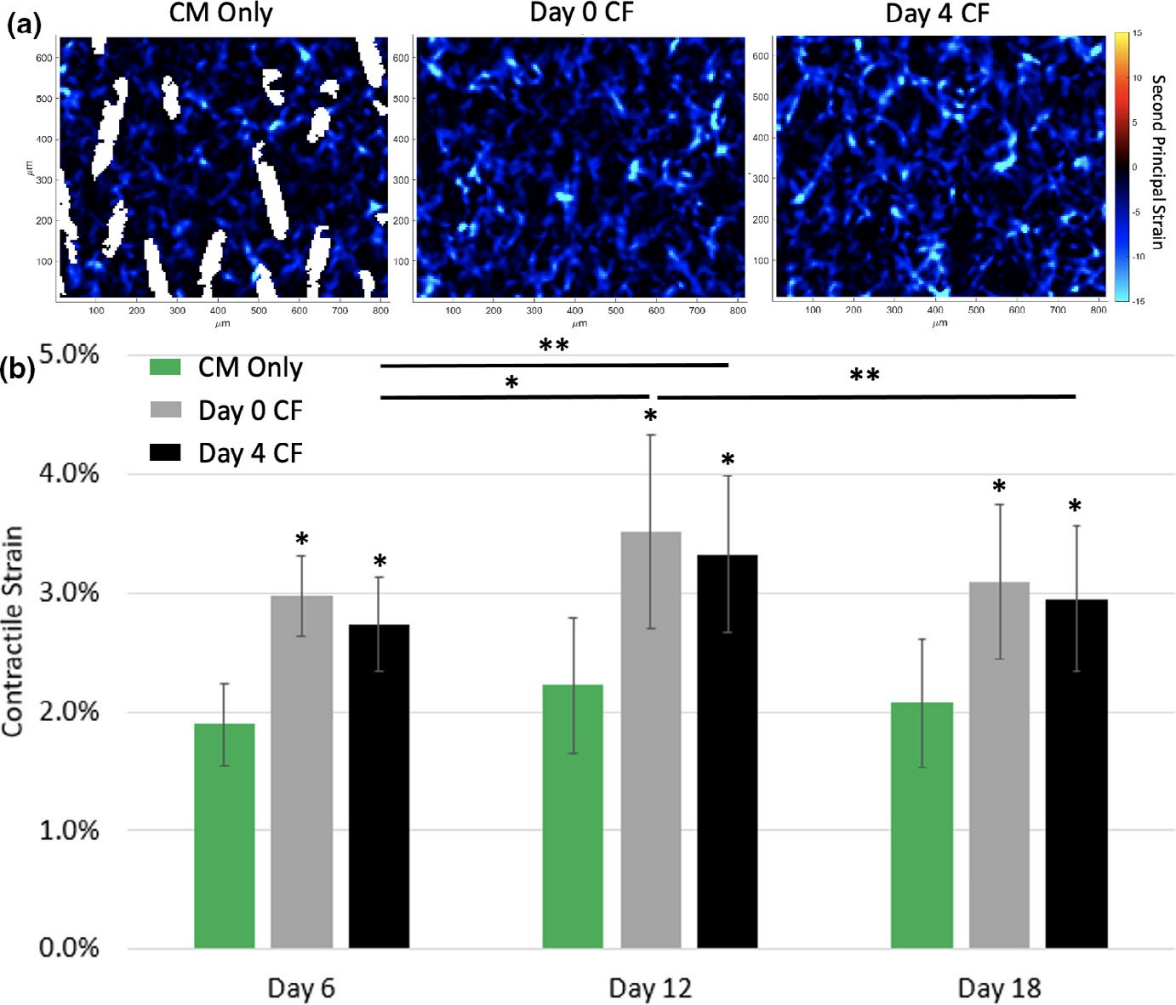
(a) Heat maps of the second principal strain for each of the three conditions during peak contraction on Day 12. (b) The maximum contractile strain for the three patterned conditions tested on Day 6, 12, and 18 of culture. *p < 0.001, **p < 0.05, two‐way ANOVA with post hoc Tukey tests. *n* = 19 for CM Only, *n* = 19 for CF Day 0, and *n* = 22 for CF Day 4. (two ROIs within a sample, 2–4 samples per experiment, three independent experiments)

After observing changes in the CaT properties of iPSC‐CMs when co‐cultured with iPSC‐CFs, we next assessed the mechanical properties of iPSC‐CMs in the micropatterned platform and used contractile strain as a measure of mechanical output. All iPSC‐CMs contracting in a 10× field of view were analyzed and the maximum average contractile strain was calculated from the full field second principal strain values for each sample (Figure 7a; Supplemental Videos 6 & 7). Repeated measures, two‐way analysis of variance (ANOVA) was performed on all samples to determine significant differences for the three conditions, number of days in culture, and strain output. For each time point tested, there was a significant difference between the maximum strain generated in the co‐culture conditions compared to the CM Only condition (*p *< 0.001; Figure 7a,b). Over the three time points tested the strain produced by the CM Only condition remained around ~2%. Likewise, there was no significant difference in strain values (3–3.5%) for the CF Day 0 condition during 18 days of culture. Strain in the CF Day 4 condition increased from 2.73 ± 0.39% at Day 6 to 3.32 ± 0.66% at Day 12 (*p *< 0.05) and remained at 2.94 ± 0.61% for Day 18. These results suggest that co‐culture with iPSC‐CFs leads to an improvement in iPSC‐CM contractile function soon after their introduction.

## 4 DISCUSSION

In order to enhance the translational prospects of iPSC‐CMs, more biomimetic models need to be developed that capture the functional electrical and mechanical behavior of iPSC‐CMs. Platforms that include individual cues stemming from the in vivo cardiac milieu such as physiologically relevant substrate stiffness (Hazeltine et al., 2012; Ribeiro et al., 2015), electrical and mechanical conditioning (Eng et al., 2016; Ronaldson‐Bouchard et al., 2018; Shadrin et al., 2017; Zhao et al., 2019), micropatterning (Napiwocki et al., 2020; Notbohm et al., 2019; Salick et al., 2014), and co‐culture (Kim et al., 2009a; Tiburcy et al., 2017) have improved iPSC‐CM function; and, the most effective platforms have the ability to combine multiple signaling factors into one in vitro culture system (Nunes et al., 2013; Ronaldson‐Bouchard et al., 2018; Ruan et al., 2016). In this work we have analyzed the structural, electrical, and mechanical influence of combined co‐culture of iPSC‐CMs with iPSC‐CFs on a soft, micropatterned substrate.

Previous studies have demonstrated that nano‐ and micro‐grooved channels influence fibroblast migration (Kim et al., 2009b; Londono et al., 2014). More recent work has demonstrated an anisotropic ECM scaffold can be generated by culturing fibroblasts on grooved PDMS surfaces followed by decellularization (Xing et al., 2014). Unlike the previous studies, we show that microscale confinement alone, with no z‐dimension topography, is sufficient to control iPSC‐CF migration and produce anisotropic fibrillar ECM spanning hundreds of micrometers. In contrast, iPSC‐CFs seeded on unpatterned monolayers were able to align the ECM locally but showed no global organization. Interestingly, we observed iPSC‐CFs remodeled the ECM more when patterned on Matrigel or fibronectin compared to the collagen I patterned ECM (Figure S4). One study reported that integrin and ECM specificity regulated extracellular signal‐regulated kinase (ERK) and c‐jun N‐ terminal kinase (JNK) differentially, with CFs activating ERK/JNK when plated on fibronectin but not when plated on collagen I (MacKenna et al., 1998). This difference in mitogen activation would alter the mechanotransduction in the CF and its ability to sense the environment leading to differences in ECM production. This work highlights the influence external cues have on cell migration and ECM organization. Future work would benefit from the investigation of decellularized anisotropic ECM scaffolds as a platform for cellular attachment.

Having characterized both iPSC‐CMs and iPSC‐CFs separately on the 10 kPa PDMS 15° chevron pattern, we next combined these cell types to produce a co‐culture model. Surprisingly, few studies have combined CMs and CFs systematically and with defined ratios (Kensah et al., 2011; Kim et al., 2009a; Miragoli et al., 2006; Tiburcy et al., 2017). In most co‐culture studies, CFs are considered part of the supporting cast necessary for matrix compaction and remodeling of 3D matrices (Boudaoud et al., 2014; Nunes et al., 2013; Ronaldson‐Bouchard et al., 2018; Tiburcy et al., 2017). Although such 3D culture techniques are capable of better replicating the cell–cell interactions found in vivo, deconvolving differences in heterogeneous cell populations are more challenging and can lead to difficulties with reproducibility and limits options for characterization. Additionally, the small cell input in this study (i.e., ~0.1 million/pattern) compared to the large number of cells (i.e., 0.5–2 million cells/construct) required for 3D studies makes this co‐culture platform more scalable for high‐throughput screening applications.

During embryonic development CFs are derived mainly from the endocardium (Wessels et al., 2012) and epicardium (Moore‐Morris et al., 2014), two distinct compartments generated after the formation of a looped heart (Furtado et al., 2016; Gittenberger‐de Groot et al., 2012). Therefore, to mimic development we patterned iPSC‐CMs on the 15° chevron pattern first and then added CFs 4 days later (Day 4 CF condition). To the best of our knowledge, this is the first co‐culture study to test the sequential addition of fibroblasts and to use iPSC‐CMs and iPSC‐CFs on soft micropatterned substrates. There is a distinct advantage to have two separate and well‐characterized iPSC‐CM and iPSC‐CF cell populations that can be combined in specific ratios and timepoints. We also consistently seeded “young” iPSC‐CMs 30 days after they were differentiated as recent reports suggest the responsiveness of iPSC‐CMs to physical stimuli declines as differentiation progresses (Ronaldson‐Bouchard et al., 2018).

In comparison to the Day 0 CF condition, there was less augmentation in the Day 4 CF condition after 18 days of culture. After decellularizing all conditions, it was found that the lane ECM in the Day 4 CF condition resembled the lane ECM in the CM Only condition (Figure S8). This led us to conclude that once iPSC‐CMs adhere to the Matrigel‐coated PDMS, iPSC‐CFs are unable to penetrate beneath the iPSC‐CMs and instead attached to the top of the iPSC‐CMs, migrate, and produce additional ECM. In comparison, the Day 0 CF condition had lane ECM in a semi‐fibrillar form indicating the iPSC‐CFs were able to remodel the ECM prior to local iPSC‐CM attachment, although not as extensively as when iPSC‐CFs were cultured alone on micropatterned lanes for 8 days.

In addition to ECM production, iPSC‐CF co‐culture is shown to influence cardiac electrophysiology. Using optical mapping, we assessed electrical impulse propagation speed and direction. Like the CM Only condition, both co‐culture conditions reported anisotropic conduction with a CV_L_/CV_T_ ratio of 2. In contrast, unpatterned iPSC‐CM monolayers (Napiwocki et al., 2020) and co‐cultures (Zhang et al., 2019) display isotropic impulse propagation and a CV_L_/CV_T_ ratio of 1. The observed anisotropy coincides with the preservation of sarcomere and myofibril alignment in the co‐culture conditions. While there exists a positive correlation between cell density and CM alignment (Badie & Bursac, 2009), the underlying 15º chevron is the main contributor of iPSC‐CM alignment in the co‐culture models presented here as augmented regions are aligned in the same orientation as the lane direction of the underlying 15º chevron pattern (Figure S7). In addition, while we report local and global orientation in the co‐culture models utilizing the 15º chevron pattern, monolayer co‐cultures do not exhibit a global orientation and thus display isotropic electrical impulse propagation (Zhang et al., 2019). These results are in good agreement with previous studies that have shown a correlation between the cardiac fiber orientation and the direction of conduction velocity (Kim et al., 2010; Roberts et al., 1979).

Importantly, there was no conduction velocity slowing in co‐culture versus the CM Only conditions, despite most prior literature showing a negative correlation with slower conduction velocity and increased percentage of CF in co‐culture (Thompson et al., 2011; Xie et al., 2009; Zhang et al., 2013). This may indicate a well‐balanced ratio of iPSC‐CM and iPSC‐CF (100:10) to maintain construct integrity without deterioration of their electrophysiological properties. An alternative explanation could be the iPSC‐CFs used in this study remain iPSC‐CFs and do not differentiate into myofibroblasts, unless treated with TGFβ1 or angiotension II (Zhang et al., 2019). It has been observed that myofibroblasts, not CFs, are responsible for fibrosis and arrhythmogenic activity where their numbers represent more than two thirds of all cardiac cell types in the diseased heart in vivo (Deb & Ubil, 2014; Nagaraju et al., 2019; Pellman et al., 2016). Conduction velocities of 10–15 cm/s reported here are on par with other 2D cardiac culture systems (Dou et al., 2020; Nunes et al., 2013).

Moreover, we find in this study that co‐culturing with iPSC‐CFs dramatically improved the CaT rise‐up time (30–37 ms) compared to the CM Only (86 ms) condition. The co‐culture values are more physiologically relevant to values of the human left ventricle myocardium (~25 ms) (Lang et al., 2015). Though not significantly different, the co‐culture conditions also had a shorter CaTD trending in the direction toward values reported for the human left ventricle (~400 ms) (Lang et al., 2015). Uniform calcium wave propagation was observed in all conditions studied. This data supports the conclusion that the co‐culture conditions had improved calcium kinetics compared to the CM Only condition.

CFs also have been shown to increase the amount of force generated by CMs in comparison to when CMs are cultured alone (Naito et al., 2006; Radisic et al., 2008; Zhang et al., 2013). In this study, we report similar findings with both co‐culture conditions producing larger contractile strains compared to the CM Only condition. While the CF Day 0 strain remained the same for the three days tested, there was an increase in strain for the Day 4 CF condition when comparing Day 6 and Day 12. This may suggest that the iPSC‐CMs need to be cultured with the iPSC‐CFs for more than 2 days before reaching a maximum contractile strain. These results highlight the importance of iPSC‐CFs in the mechanical function of iPSC‐CMs.

## 5 CONCLUSION

While CMs make the tissue of the heart contract, they are not the only cell type present in the native myocardium. Here, we demonstrate the importance of CFs and their role in ECM production and maintenance. When cultured alone on soft micropatterned substrates, iPSC‐CFs are confined to the micropatterned features and remodel the ECM into anisotropic fibers. We show similar remodeling and ECM production occurs when iPSC‐CFs are co‐cultured with iPSC‐CMs. In addition to ECM, our results indicate that iPSC‐CFs influence iPSC‐CM function with improved calcium kinetics and greater contractile strains in the co‐culture conditions compared to when iPSC‐CMs are cultured alone. These combined observations highlight the important role of CFs in vivo and incorporation of CFs in a co‐culture model will allow for more accurate in vitro cardiac constructs.

## CONFLICTS OF INTEREST

No conflict of interest, financial or otherwise, is declared by the author.

## AUTHOR CONTRIBUTIONS

LLE, AVG, TJK, and WCC contributed to the conception and funding acquisition for the study. BNN, JZ, TJK, and WCC contributed to methodological development of the study. BNN, AS, DL, and GCK had primary responsibility for conducting the experiments, data acquisition, curation, and analysis, with assistance from JZ and RAK. TJK and WCC had lead roles in project oversight. BNN drafted the manuscript, with assistance from TJK and WCC. All authors edited and reviewed the manuscript.

## Supporting information



Fig S1

Fig S2

Fig S3

Fig S4

Fig S5

Fig S6

Fig S7

Fig S8

Fig S9

Fig S10

Movie S01

Movie S02

Movie S03

Movie S04

Movie S05

Video S06

Video S07
