# Supplementary figures and images for: Micropattern platform promotes extracellular matrix remodeling by human PSC‐derived cardiac fibroblasts and enhances contractility of co‐cultured cardiomyocytes
Source: Physiol Rep. 2021 Oct 7;9(19):e15045. doi: 10.14814/phy2.15045 (PMC8496154; doi:10.14814/phy2.15045)

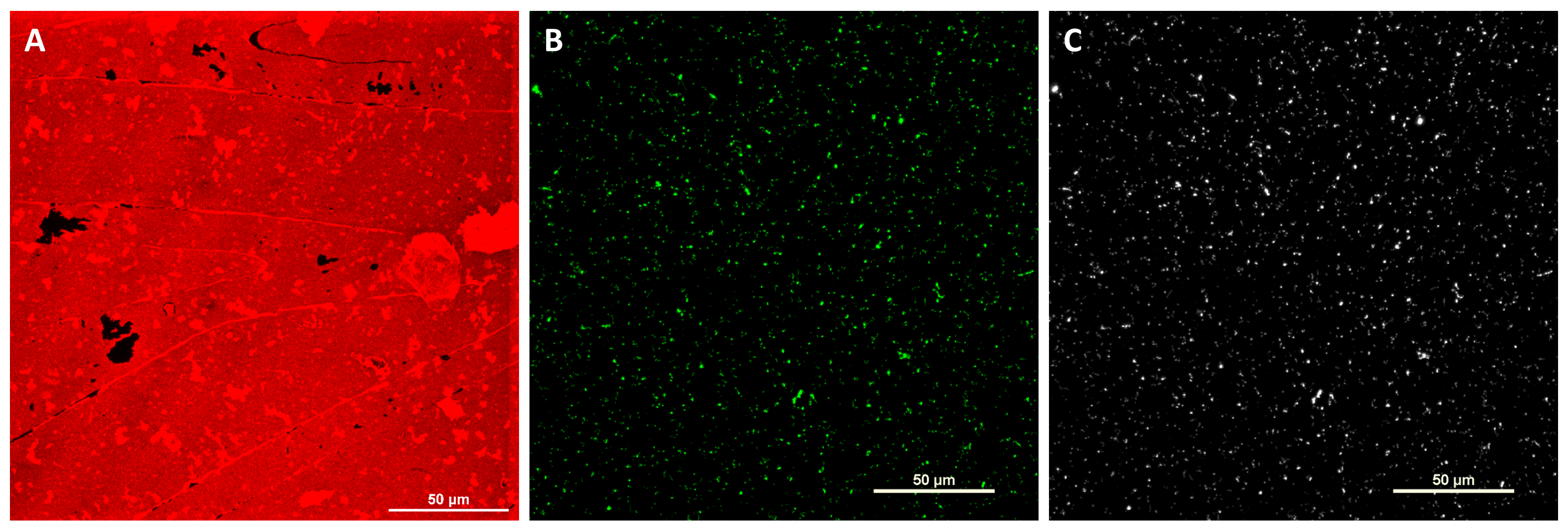

Supplement: Supplementary file 1 — Fig S1 [file PHY2-9-e15045-s008.tif]

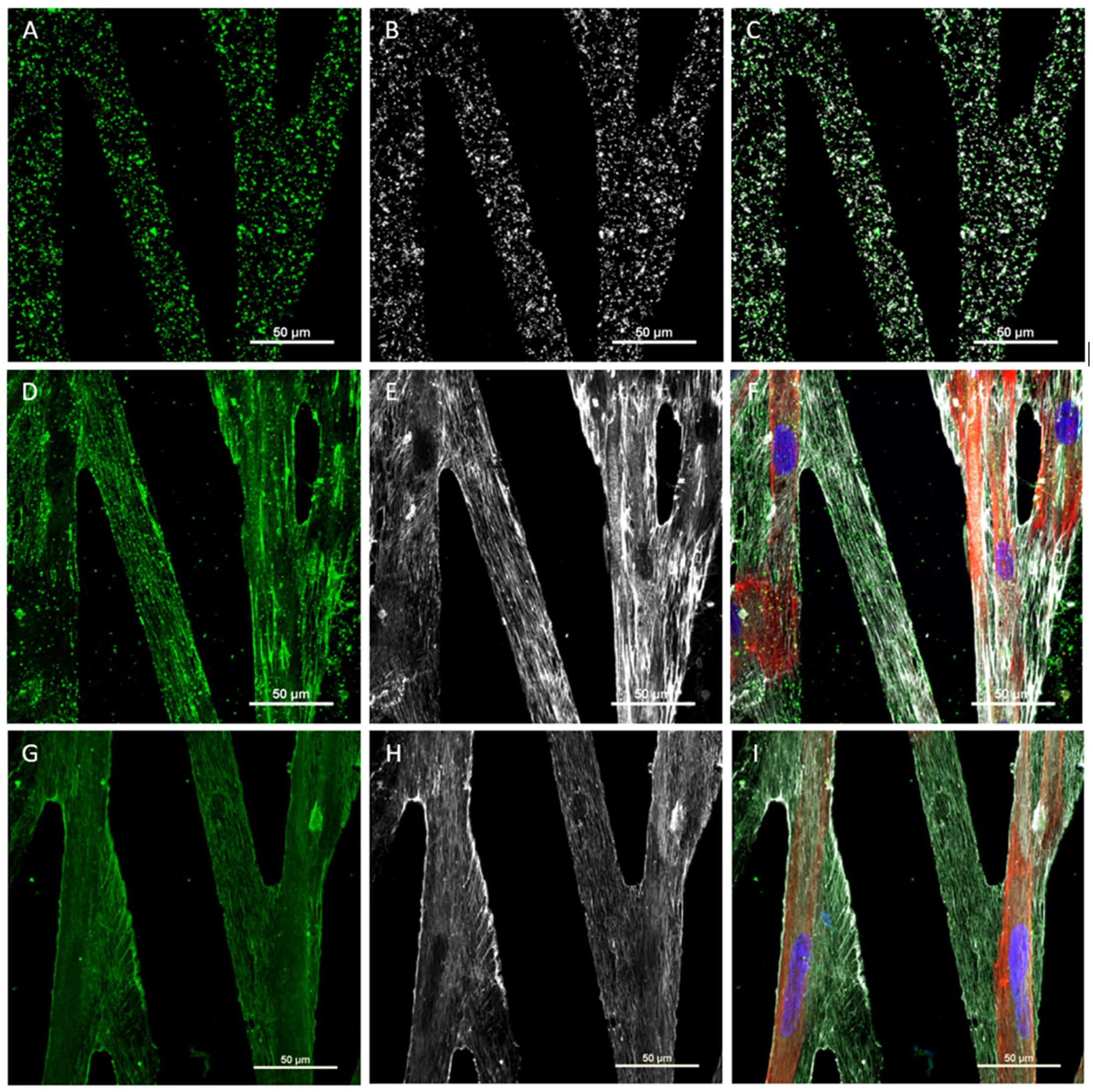

Supplement: Supplementary file 2 — Fig S2 [file PHY2-9-e15045-s015.tiff]

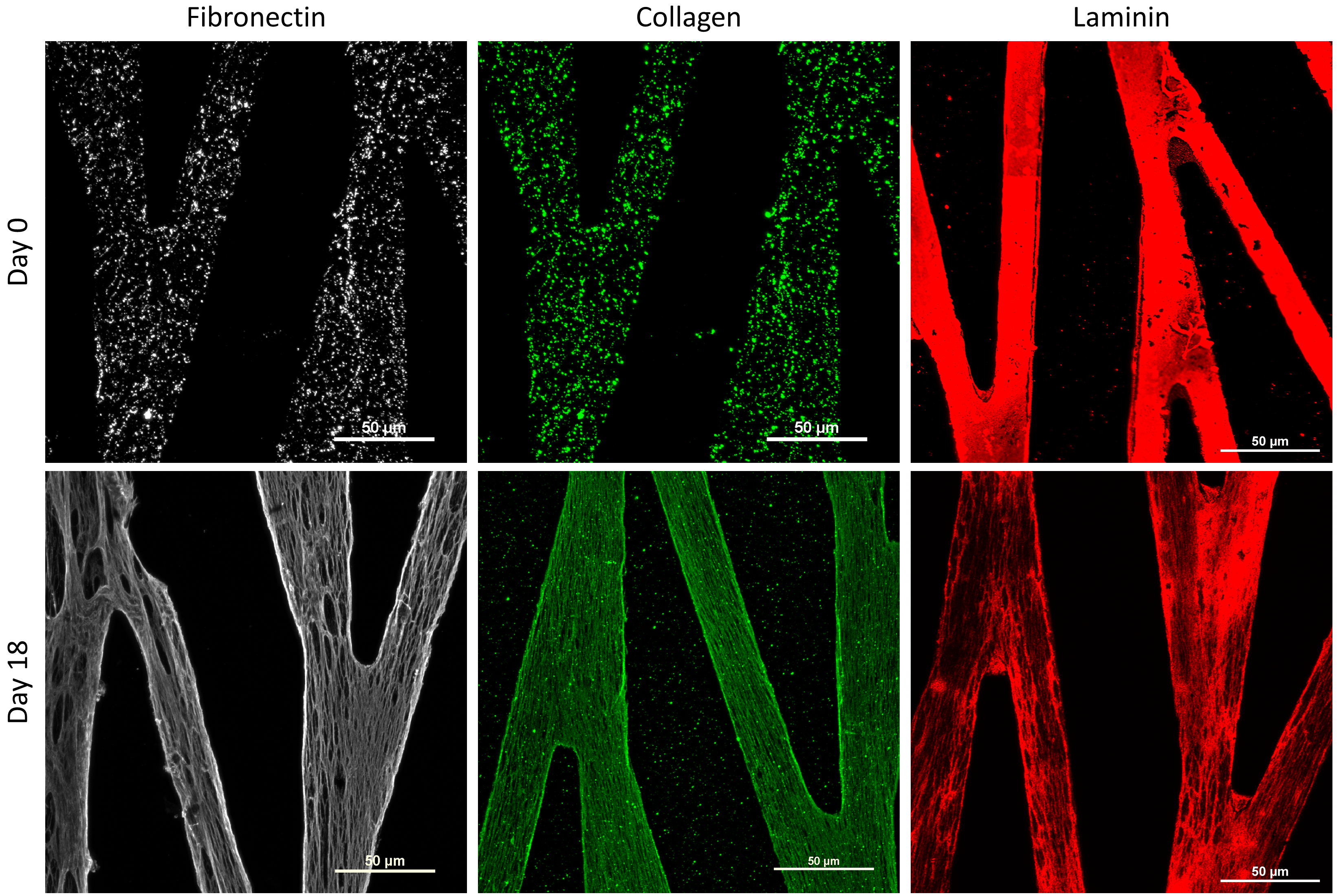

Supplement: Supplementary file 3 — Fig S3 [file PHY2-9-e15045-s005.tif]

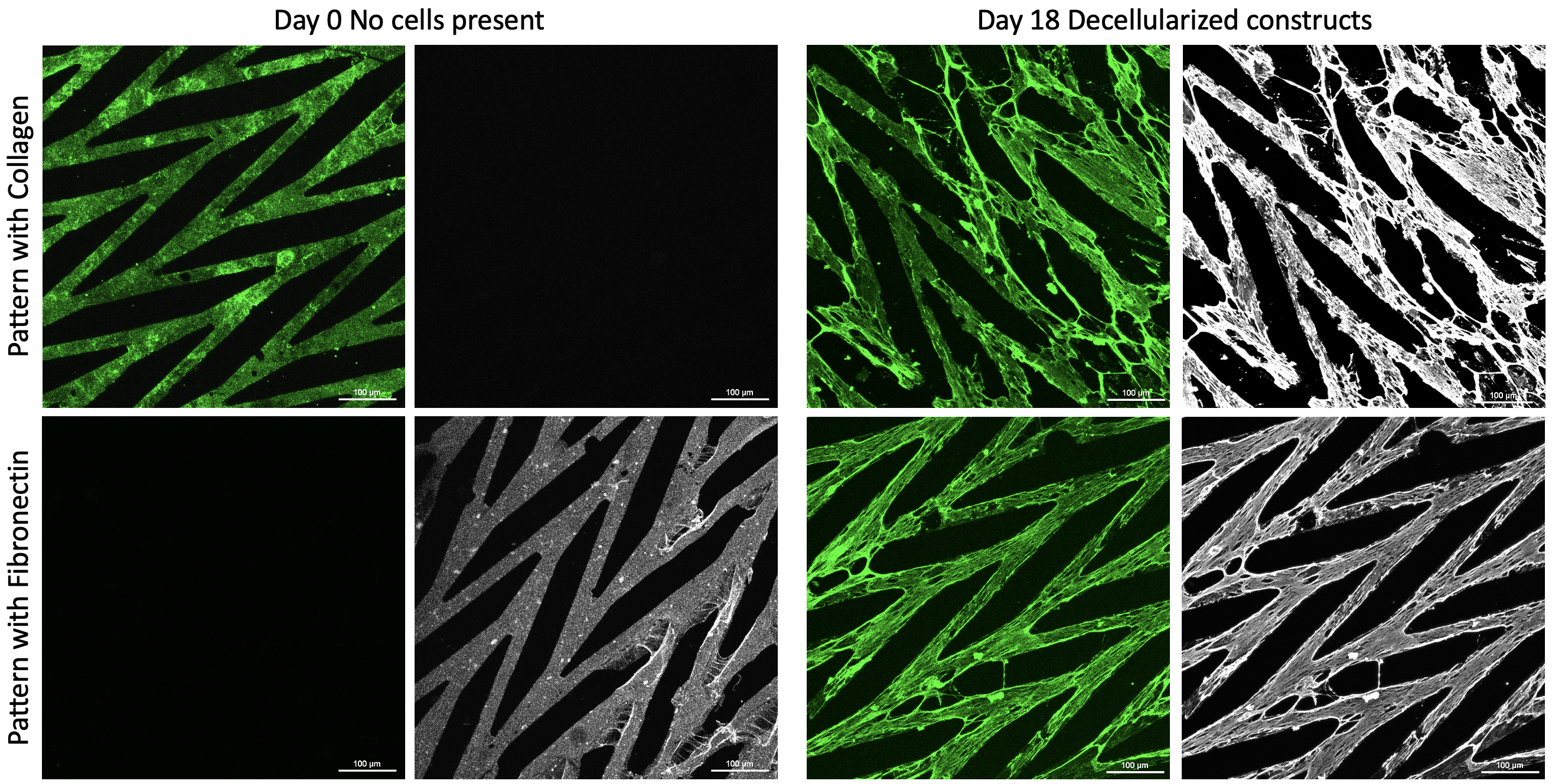

Supplement: Supplementary file 4 — Fig S4 [file PHY2-9-e15045-s017.png]

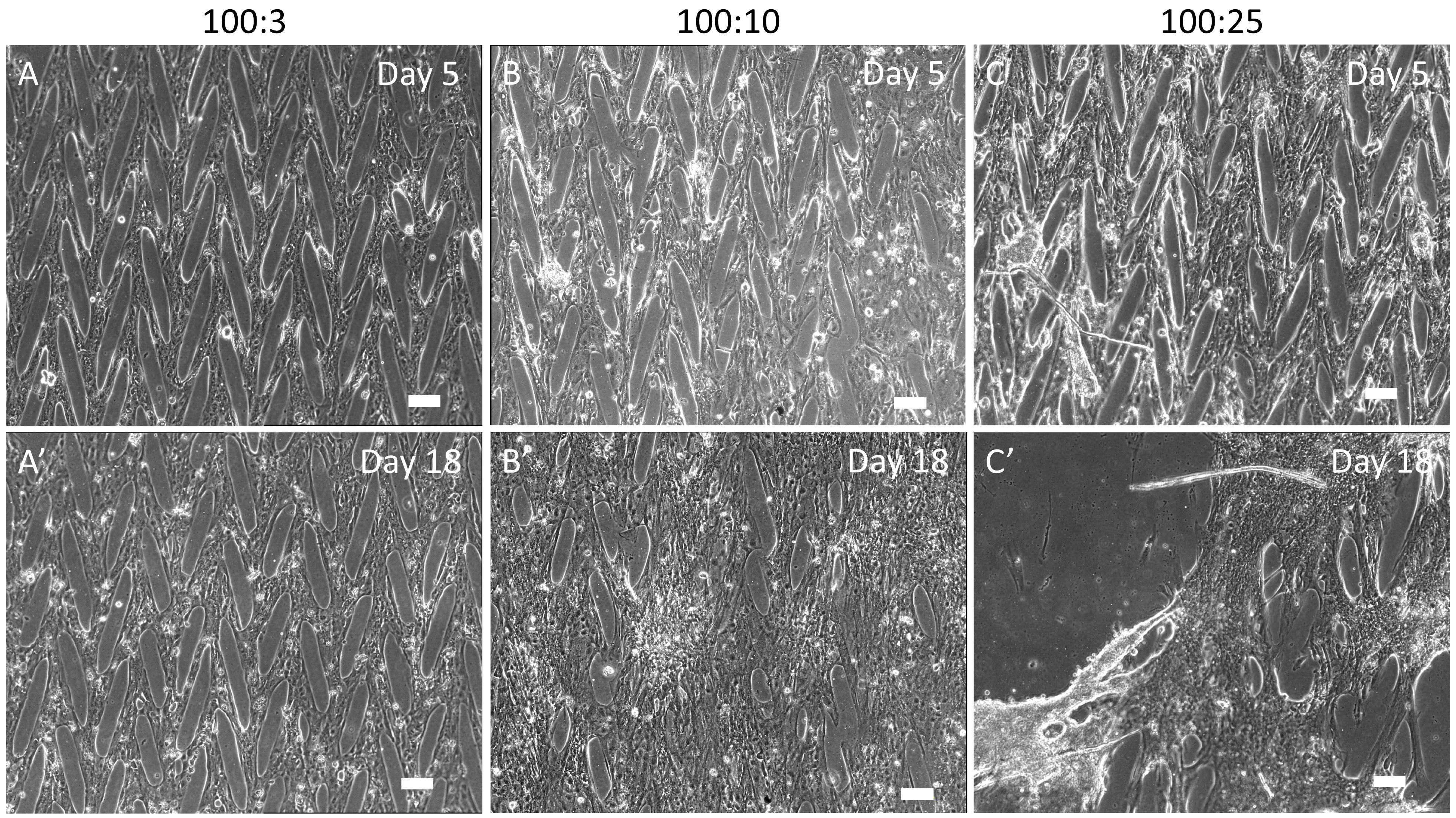

Supplement: Supplementary file 5 — Fig S5 [file PHY2-9-e15045-s010.tif]

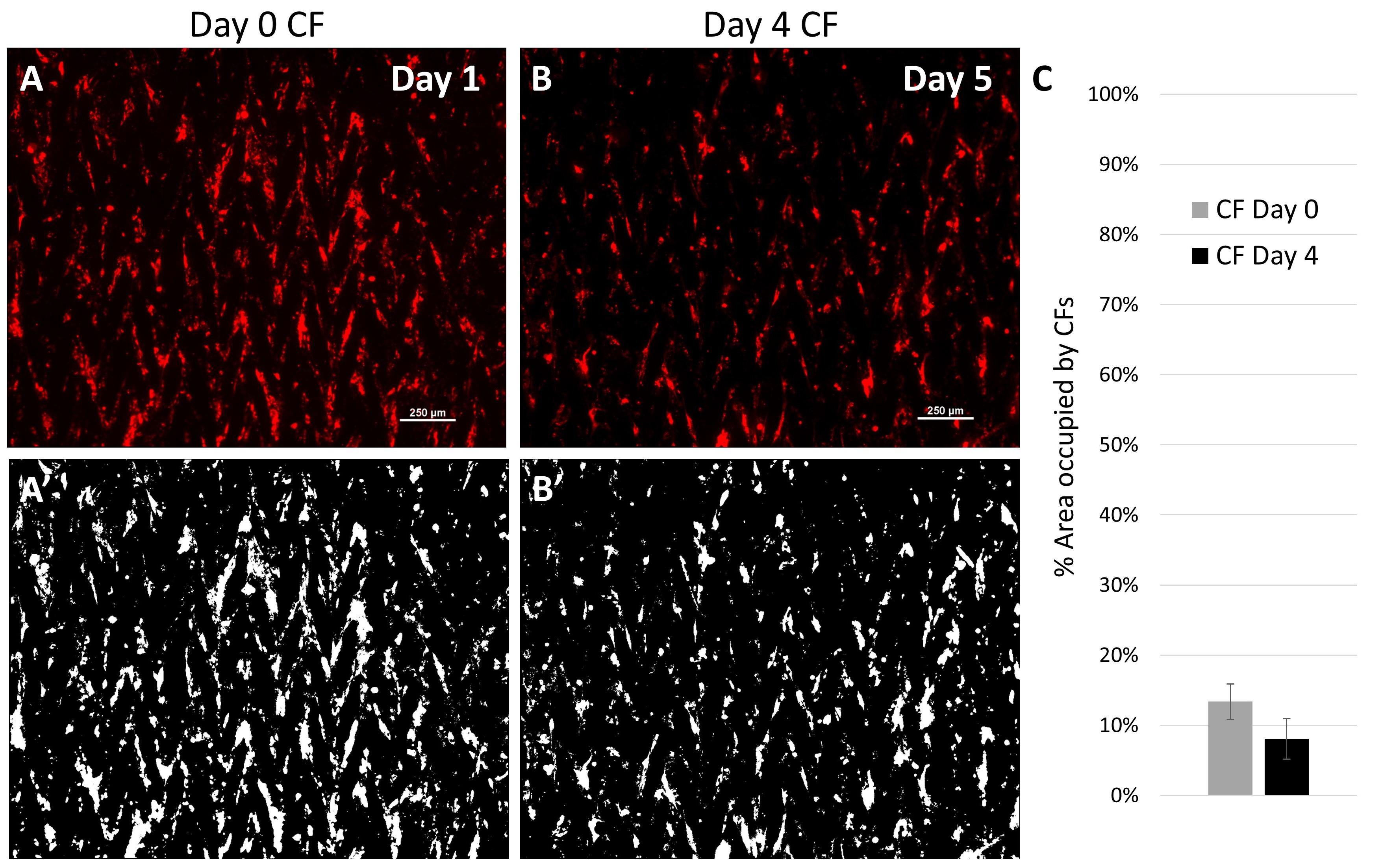

Supplement: Supplementary file 6 — Fig S6 [file PHY2-9-e15045-s013.tiff]

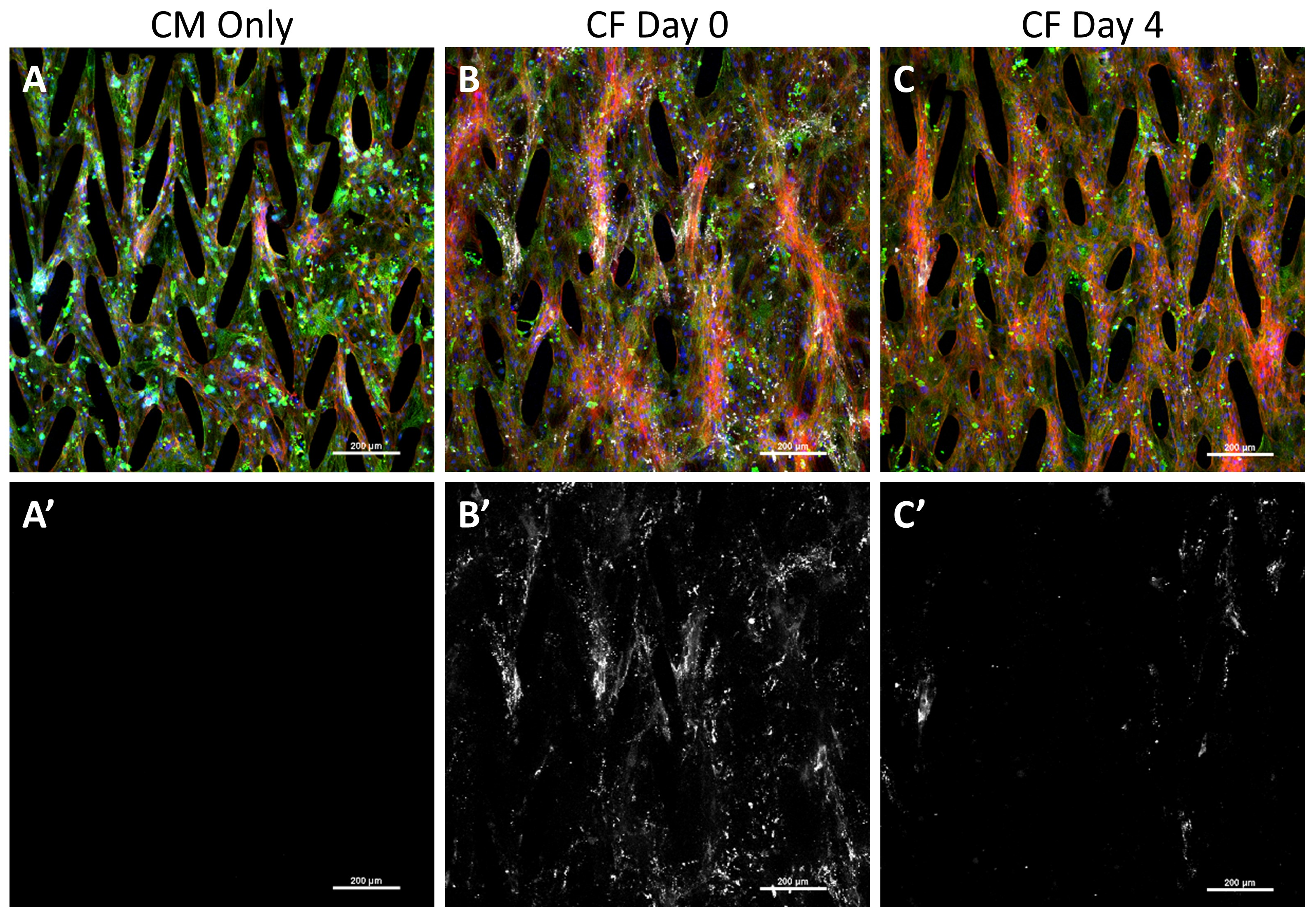

Supplement: Supplementary file 7 — Fig S7 [file PHY2-9-e15045-s009.tiff]

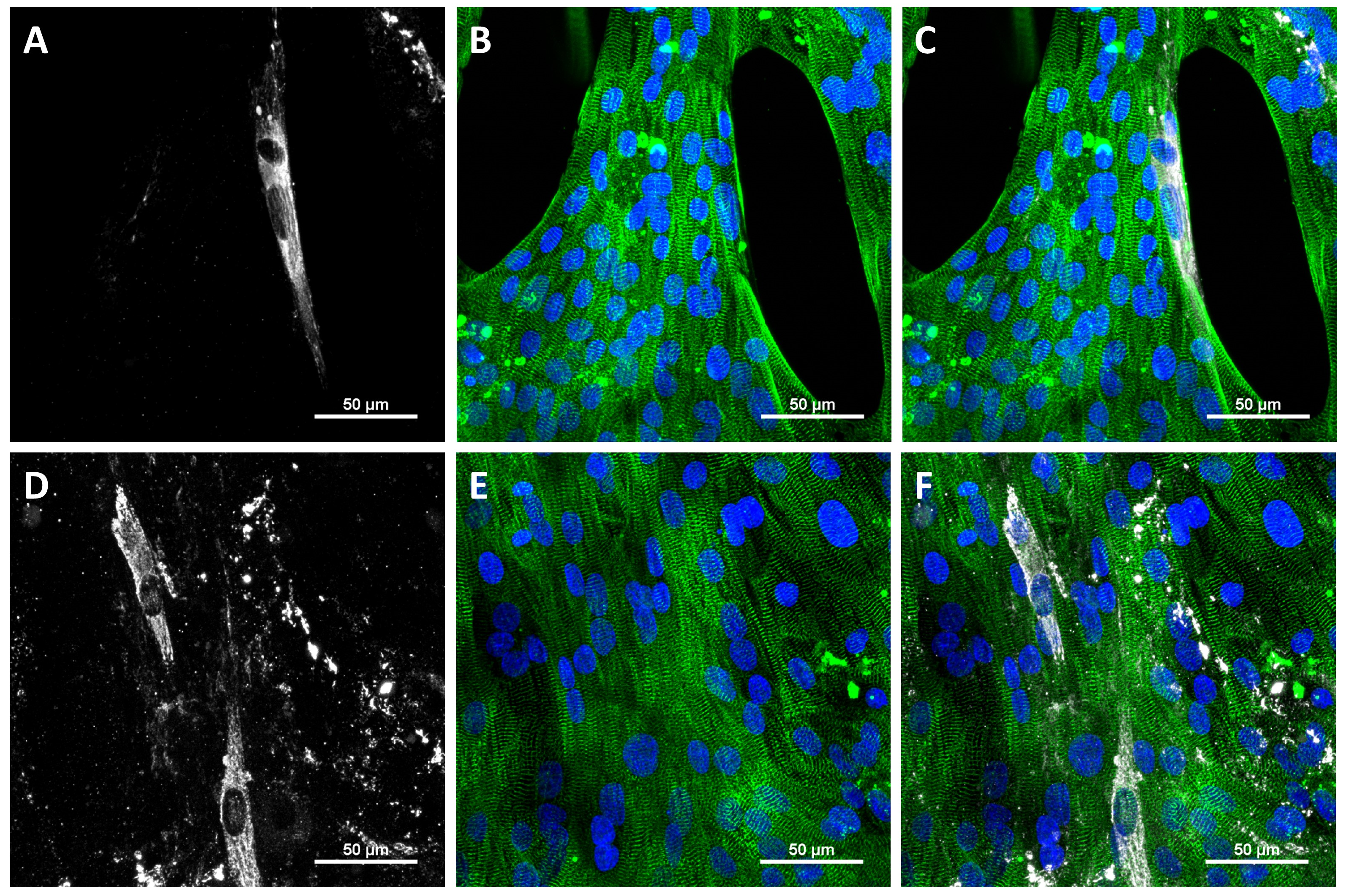

Supplement: Supplementary file 8 — Fig S8 [file PHY2-9-e15045-s003.tiff]

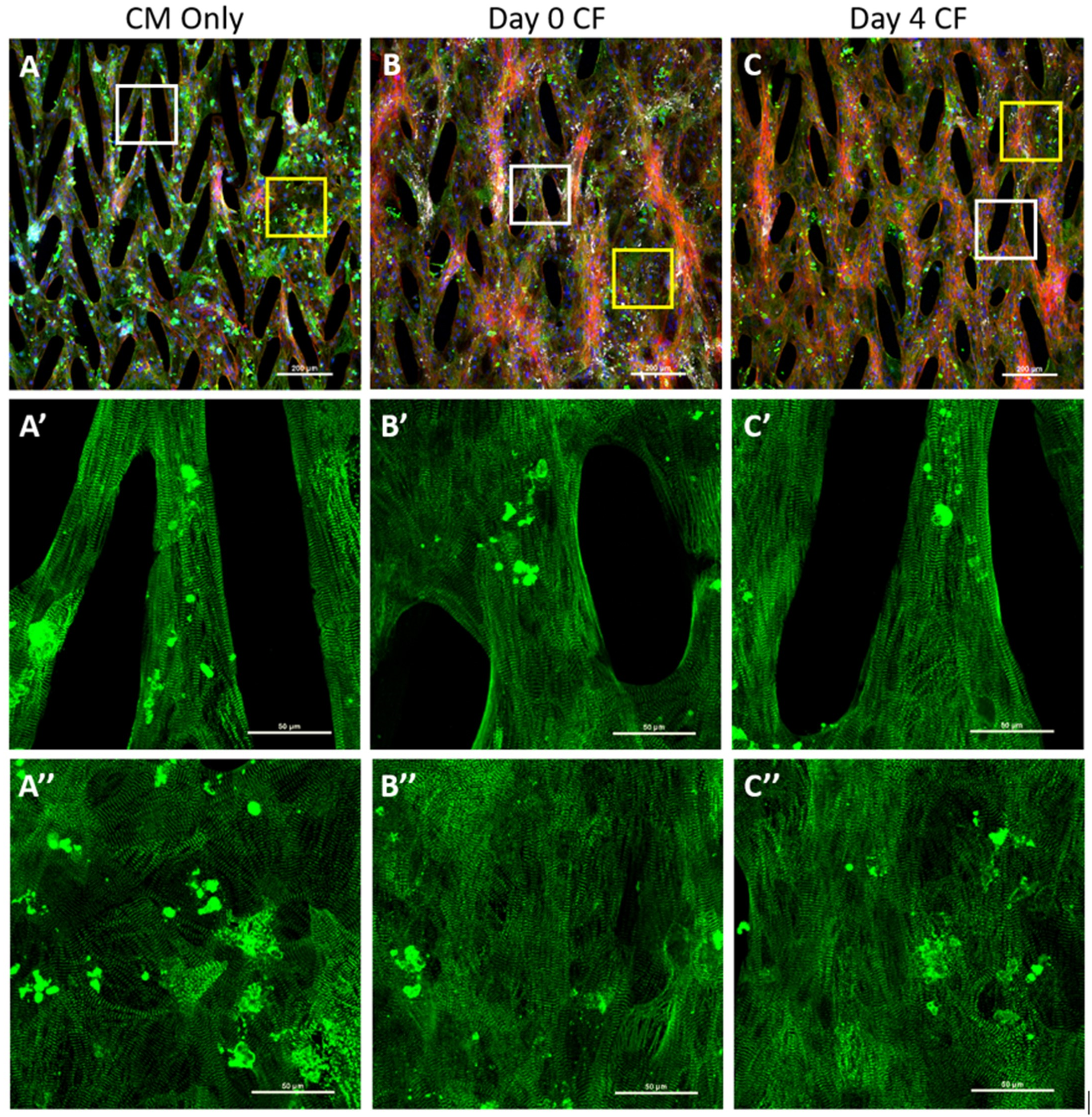

Supplement: Supplementary file 9 — Fig S9 [file PHY2-9-e15045-s012.tiff]

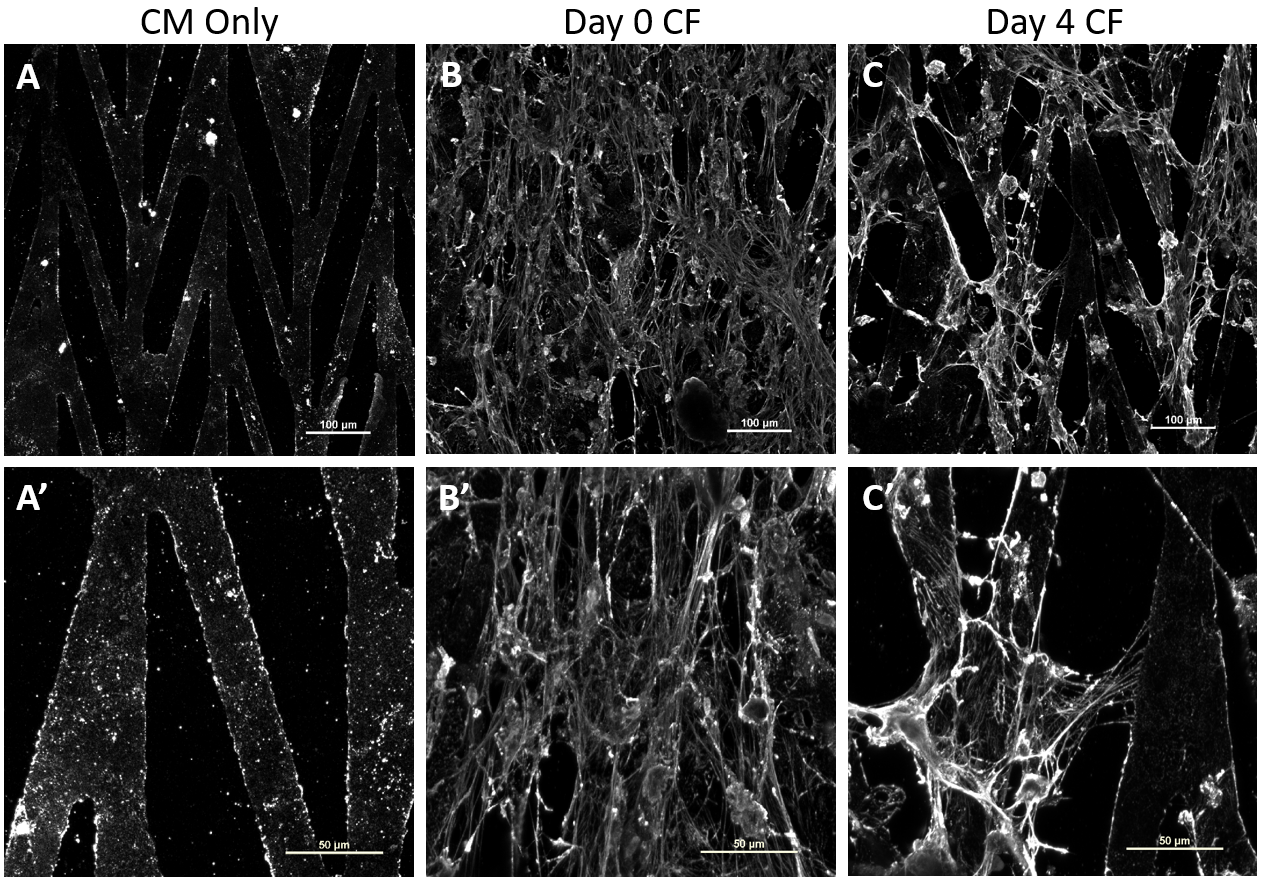

Supplement: Supplementary file 10 — Fig S10 [file PHY2-9-e15045-s004.tif]
